# Cell Type-Selective Loss of Peroxisomal β-Oxidation Impairs Bipolar Cell but Not Photoreceptor Survival in the Retina

**DOI:** 10.3390/cells11010161

**Published:** 2022-01-04

**Authors:** Daniëlle Swinkels, Yannick Das, Sai Kocherlakota, Stefan Vinckier, Eric Wever, Antoine H.C. van Kampen, Frédéric M. Vaz, Myriam Baes

**Affiliations:** 1 Laboratory of Cell Metabolism, Department of Pharmaceutical and Pharmacological Sciences, KU Leuven, 3000 Leuven, Belgium; danielle.swinkels@kuleuven.be (D.S.); yannick_das@hotmail.com (Y.D.); sai.kocherlakota@kuleuven.be (S.K.); 2 Laboratory of Angiogenesis and Vascular Metabolism, Department of Oncology, VIB, 3000 Leuven, Belgium; stefan.vinckier@kuleuven.be; 3 Bioinformatics Laboratory, Department of Epidemiology and Data Science, Amsterdam Public Health Research Institute, Amsterdam University Medical Center (UMC), University of Amsterdam, 1100 DE Amsterdam, The Netherlands; eric.wever@amsterdamumc.nl (E.W.); a.h.vankampen@amsterdamumc.nl (A.H.C.v.K.); 4 Biosystems Data Analysis, Swammerdam Institute for Life Sciences, University of Amsterdam, 1090 GE Amsterdam, The Netherlands; 5 Core Facility Metabolomics, Amsterdam University Medical Center (UMC), 1105 AZ Amsterdam, The Netherlands; f.m.vaz@amsterdamumc.nl; 6 Laboratory of Genetic Metabolic Diseases, Department of Clinical Chemistry and Pediatrics, Amsterdam University Medical Center (UMC), University of Amsterdam, 1105 AZ Amsterdam, The Netherlands

**Keywords:** peroxisome, β-oxidation, multifunctional protein 2, PEX5, photoreceptor, bipolar cell, PUFA, VLCFA, VLC-PUFA, retina

## Abstract

Retinal degeneration is a common feature in peroxisomal disorders leading to blindness. Peroxisomes are present in the different cell types of the retina; however, their precise contribution to retinal integrity is still unclear. We previously showed that mice lacking the central peroxisomal β-oxidation enzyme, multifunctional protein 2 (MFP2), develop an early onset retinal decay including photoreceptor cell death. To decipher the function of peroxisomal β-oxidation in photoreceptors, we generated cell type selective *Mfp2* knockout mice, using the *Crx* promotor targeting photoreceptors and bipolar cells. Surprisingly, *Crx-Mfp2^−/−^* mice maintained photoreceptor length and number until the age of 1 year. A negative electroretinogram was indicative of preserved photoreceptor phototransduction, but impaired downstream bipolar cell signaling from the age of 6 months. The photoreceptor ribbon synapse was affected, containing free-floating ribbons and vesicles with altered size and density. The bipolar cell interneurons sprouted into the ONL and died. Whereas docosahexaenoic acid levels were normal in the neural retina, levels of lipids containing very long chain polyunsaturated fatty acids were highly increased. *Crx-Pex5^−/−^* mice, in which all peroxisomal functions are inactivated in photoreceptors and bipolar cells, developed the same phenotype as *Crx-Mfp2^−/−^* mice. In conclusion, the early photoreceptor death in global *Mfp2^−/−^* mice is not driven cell autonomously. However, peroxisomal β-oxidation is essential for the integrity of photoreceptor ribbon synapses and of bipolar cells.

## 1. Introduction

Peroxisomes are involved in many processes, including reactive oxygen species (ROS) regulation, cellular stress response and antiviral defense [1,2]. They are also highly metabolic active cell organelles and they have a crucial role in lipid metabolism via peroxisomal β-oxidation [1]. Patients with a mutation in the central enzyme of this pathway, multifunctional protein 2 (MFP2), often present with retinopathy, resulting in blindness [3,4,5,6,7]. MFP2, also called D-bifunctional protein (DBP), is encoded by *Hsd17b4*, and catalyzes the second and third step of peroxisomal β-oxidation. The potential importance of peroxisomal β-oxidation for the retina is further supported by the fact that it is involved in the metabolism of principal components of the retina. It catabolizes various substrates, including very long chain polyunsaturated fatty acids (VLC-PUFAs). Furthermore, one cycle of peroxisomal β-oxidation is necessary to synthesize docosahexaenoic acid (DHA, C22:6n−3) [8]. Both of these lipid species are highly enriched in the photoreceptor outer segments (POS), where they play a role in the phototransduction process [9,10,11,12,13]. 

In the retina, peroxisomes are abundant in the retinal pigment epithelium (RPE), in photoreceptors and ganglion cells, and to a lesser extent in the outer (OPL) and inner plexiform layer (IPL) [14,15,16,17,18]. Furthermore, in situ hybridization revealed that *Mfp2* mRNA is prominently present in the RPE and photoreceptor inner segments (PIS), but transcripts were also observed in the outer (ONL) and inner nuclear layer (INL), OPL and ganglion cell layer [19]. We recently showed that a mouse model lacking MFP2 (*Mfp2^−/−^*) develops early onset retinal degeneration, underscoring the importance of peroxisomal β-oxidation for retinal integrity [19]. These *Mfp2^−/−^* mice presented with impaired visual function at 3 weeks (w) and reduced visual acuity at 8 w. Furthermore, POS were already shortened at 2 w and progressive photoreceptor degeneration was seen. This was accompanied by a significant reduction in DHA containing phospholipid species in the retina [19]. In addition, several anomalies developed in the RPE. In view of the intricate interrelation between photoreceptors and RPE cells, it was difficult to pinpoint the mechanism underlying the retinal degeneration in global *Mfp2^−/−^* mice. Furthermore, the specific role of MFP2 in the different retinal cell types remained unexplored. The need to elucidate the role of peroxisomes and of peroxisomal β-oxidation in the retina has become even more critical, since currently gene therapy to alleviate the retinal symptoms of peroxisome-deficient patients is being developed [20]. 

Since it has been shown before that peroxisomes are highly present in photoreceptors [14,15,16,17,18] and that they can locally synthesize DHA [21,22,23,24], the question arose whether the lack of peroxisomal β-oxidation within photoreceptors is crucial for the phenotype of *Mfp2^−/−^* mice. Therefore, we generated cell type selective *Mfp2* knockout mice (*Crx-Mfp2^−/−^* mice), using the cone-rod homeobox (*Crx*) promotor [25]. Of note, the *Crx* promotor targets rods, cones and bipolar cells [25]. As the retinal phenotype was surprisingly mild in the *Crx-Mfp2^−/−^* mice and peroxisomes might exert other metabolic functions in photoreceptors, we subsequently created *Crx-Pex5^−/−^* mice in which the entire peroxisome was inactivated. PEX5 is the receptor required for the import of matrix proteins into peroxisomes, and its absence causes a complete inactivation of functional peroxisomes [26,27,28]. 

Using in vivo functional tests and (immuno) histochemistry, we here show that photoreceptor integrity and function was maintained in both studied mouse models with the exception of the photoreceptor ribbon synapse, while bipolar cell function and integrity was affected. Lipidomic analyses revealed that this was not associated with a lack of DHA, but that lipids containing VLC-PUFAs strongly accumulated. These findings suggest that the early photoreceptor death in global *Mfp2^−/−^* mice is not driven cell autonomously. However, we can conclude that peroxisomes are essential for the signaling between photoreceptors and bipolar cells, whereby both the ribbon synapse and the receiving cells are affected.

## 2. Materials and Methods

### 2.1. Generation of Crx-Mfp2^−/−^ and Crx-Pex5^−/−^ Mice 

To generate conditional *Mfp2* knockout mice, *Crx-Cre* [20] mice, kindly provided by T. Glaser, were used. These mice express Cre recombinase under the control of the *Crx* promotor, causing rod, cone and bipolar cell-specific deletion starting at the embryonic stage (E13.5) [25]. In the first round of breeding *Crx-Cre* mice were crossed with *Mfp2^L/L^* mice [29], generating *Crx-Cre Mfp2^WT/L^* mice. In the second generation, the selected *Crx-Cre Mfp2^WT/L^* mice were crossed once more with *Mfp2^L/L^* mice, resulting in *Crx-Cre Mfp2^L/L^* mice, herein denoted as *Crx-Mfp2^−/−^* mice. As no morphological or functional abnormalities were observed, mice with one of the other possible genotypes (*Mfp2^WT/L^*, *Mfp2^L/L^* and *Crx-Mfp2^WT/L^*) were considered control mice. *Crx-Pex5^−/−^* mice were generated in a similar manner using *Pex5^L/L^* mice [30]. Polymerase chain reaction (PCR) analysis was conducted to identify animals with the genotype of interest (primers are listed in Table 1).

All animals were bred in the conventional animal housing facility of the KU Leuven, were kept on a 13–11 h light and dark cycle and had ad libitum access to water and standard rodent food. All experiments were in accordance with the Guidelines for Care and Use of Experimental Animals (NIH) and were fully approved by the Research Ethical Committee of the KU Leuven (P166/2017). For all experiments, except the optokinetic tracking response test, animals were deeply anaesthetized with a mix of Nimatek (75 mg/kg) and Domitor (1 mg/kg), followed by cervical dislocation to sacrifice the mice.

### 2.2. In Vivo Analysis of Visual Acuity and Functioning 

Visual acuity was assessed using the optokinetic tracking response, as previously described [31,32]. Briefly, unrestrained mice were placed on an elevated platform and subjected to a stimulus consisting of vertical sine-wave gratings (black/white) at a speed of 12°/second. When this pattern is detected by the mouse, a head movement in the same direction as the stimulus can be observed. The spatial frequency was gradually increased to the point where the mouse no longer responded, determining the maximum spatial frequency. The spatial frequency was assessed for both eyes and the average was taken. 

To measure visual functionality, full-field electroretinogram (ERG) was performed using the Celeris system (Diagnosys). Firstly, mice were overnight dark adapted, followed by anesthesia and dilation of the pupils with 0.5% tropicamide (Tropicol, Thea Pharma) and 15% phenylephrine (Thea Pharma). To keep the eyes moist and to conduct the electrical signal, Genteal drops (Novartis) were added. The scotopic ERG response (rod-mediated response) was determined by applying increasing flash intensities ranging from 0.01 to 8 cd*s/m^2^. Subsequently, eyes were light adapted by subjecting to light for 10 min at 9 cd*s/m^2^. The photopic response (cone-mediated) was then measured at 3 and 10 cd*s/m^2^. The software calculated the a- and b-wave amplitudes, representing the photoreceptor and interneuron cell response, respectively. The a-wave amplitude was quantified from baseline to the trough of the negative peak, while the b-wave was calculated from the trough of the negative peak to the crest of the positive peak. 

Flicker ERG responses were measured at a fixed luminance (0.5 log cd*s/m^2^) with increasing frequency (0.5, 1, 5, 10, 20 and 30 Hz), where the rod bipolar cell response is measured below 5 Hz and the cone bipolar cell response above 5 Hz [33,34]. Responses were quantified from the through of the negative peak to the crest of the positive peak. 

### 2.3. Western Blotting

Samples were prepared as previously described [19]. Protein concentrations were measured using the Pierce BCA protein kit (Thermo Scientific, Rockford, IL, USA), according to the manufacturer’s instructions. Samples were separated on a 10% polyacrylamide gel, the proteins were transferred onto a polyvinylidine fluoride membrane and blocked with blocking buffer (5% (*w*/*v*), defatted milk powder in Tris Buffered Saline with 0.1% (*v*/*v*) Tween20). Next, membranes were overnight incubated at 4 °C with primary antibodies (Table 2), followed by probing with the appropriate horseradish peroxidase (HRP)-conjugated secondary antibody (Dako, Glostrup, Denmark) (1/5000). To visualize the bands, Amersham ECL Western Blotting Detection Reagent (GE Healthcare Life Science, Buckinghamshire, UK) was added to the membranes and the ChemiDoc MP System (Bio-Rad, Hercules, CA, USA) was used. Images were processed with the Image Lab software (Bio-Rad). Vinculin served as loading control.

### 2.4. Lipidome 

Lipidome analysis was performed as previously described [36]. Briefly, mice were deeply anesthetized, after which the neural retina was isolated as explained [19]. Subsequently, the neural retina was homogenized by sonication in 60 µL medium (250 mM sucrose, 5 mM MOPS (pH 7.2), 1 mM EDTA and 0.1% (*v*/*v*) ethanol) and protein concentration was determined with the Pierce BCA protein kit. Next, internal standards were added to the homogenates and phospholipids were extracted with a single-phase extraction using chloroform/methanol (1:1, *v*/*v*). The extracted lipids were separated using both normal phase and reverse phase high-performance liquid chromatography, linked to mass spectrometry in positive and negative electrospray ionization mode. The obtained datasets were processed using an in-house developed metabolomics pipeline [36], which included identification of the peaks, isotope correction, normalization to the internal standards and statistical analysis. Of note, comparisons can only be done between different groups (CT vs. *Crx-Mfp2^−/−^*) within the same lipid species. Data are presented as fold change compared to CT levels. 

### 2.5. Histochemistry 

Enucleated eyes were fixed overnight at 4 °C in either New Davidson’s Fixative (NDF) (22.2% (*v*/*v*) formaldehyde 10%, 32% (*v*/*v*) alcohol, 11.1% (*v*/*v*) glacial acetic acid) or 1% paraformaldehyde (PFA), embedded in paraffin and cut in 7 µm thick transverse retinal sections. To assess gross morphology, Hematoxylin–Eosin (HE) staining was carried out on NDF sections. Images were acquired with an inverted IX-81 microscope (Olympus, 20× objective: UPLFLN20×). Measurement of the length of the photoreceptor layer (PR), photoreceptor outer segments (POS) and photoreceptor inner segments (PIS) was performed with phase contrast microscopy. NDF-fixed eyes were deparaffinized in xylene (100%), followed by mounting with dibutyl phthalate in xylene (DPX, Darmstadt, Germany). Per mouse, one image was taken on either side of the optic nerve (nasal and temporal), on which three measurements were executed, using ImageJ (NIH). The average value from the two planes was taken. Images were acquired with a Leica DMI6000B microscope, using phase-contrast settings (63× objective: HCX PL Fluotar 63×/1.25 oil PH3).

### 2.6. Immunohistochemistry 

Immunohistochemical (IHC) staining was performed as previously described [14,19]. In short, NDF sections were deparaffinized and rehydrated, followed by antigen retrieval by heating the sections for 10 min in 1 mM EDTA (pH 8.0). Next, endogenous peroxidases were inactivated in 3% (*v*/*v*) H_2_O_2_, in case an HRP-conjugated antibody was used. To reduce non-specific binding, sections were blocked with 2% (*v*/*v*) normal goat serum in blocking buffer (0.1 M Tris-HCl pH 7.5, 0.15 M NaCl, 0.5% (*w*/*v*) blocking reagent (Perkin Elmer, Boston, USA), followed by overnight incubation at 4 °C with primary antibodies (Table 2). Next, either the appropriate HRP-conjugated IgG (1/200) (Agilent) or Alexa Fluor IgG-coupled secondary antibody (1/200) (Invitrogen, Eugene, OR, USA) was added. In case an HRP-conjugated antibody was applied, the fluorescein TSA plus amplification kit (Perkin Elmer, Boston, MA, USA) was used according to the manufacturer’s instructions. Lastly, sections were counterstained with Hoechst 33342 (Sigma, St. Louis, MO, USA) and mounted with Prolong^®^ Gold antifade mountant (Invitrogen, Eugene, OR, USA). Images were acquired with a Leica SP8× confocal microscope.

To visualize Cre recombinase, enucleated eyes were fixed overnight at 4 °C in 4% PFA. Afterwards, eyes were submerged in increasing concentrations of sucrose (10, 20 and 30% (*w*/*v*)), followed by embedding in optimum cutting temperature Tissuetek (OCT, Thermo Scientific, Runcorn, UK). Thawed sections were first blocked and then incubated overnight at 4 °C with Cre recombinase antibodies, followed by incubation with Alexa Fluor 568 goat anti-mouse IgG. Lastly, sections were counterstained with Hoechst 33342 and mounted.

Protein kinase Cα (PKCα)/ vesicular glutamate transporter 1 (VGLUT1) double staining and 4-hydroxy-2-nonenal (4-HNE) staining was performed on 1% PFA paraffin sections. After deparaffinization and rehydration, antigen retrieval was performed. For PKCα/VGLUT1 double staining, antigen retrieval was executed using the reagents from the RNAScope^®^ 2.5 HD Chromogenic Detection Kit (see below), according to the manufacturer’s protocol. Antigen retrieval for 4-HNE staining was performed by heating in 0.1 M citrate buffer (pH 6.0), as previously described [14], followed by inactivation of endogenous peroxidases with 3% (*v*/*v*) H_2_O_2_. Next, sections were blocked with blocking buffer and overnight incubated at 4 °C with primary antibodies. Afterwards, sections were probed with Alexa fluor IgG coupled secondary antibodies (1/200) or with HRP-conjugated IgG (1/200), combined with the fluorescein TSA plus amplification kit, counterstained with Hoechst 33342 and mounted. The number of PKCα positive bipolar cells were counted over a distance of 100 µm at both sides of the optic nerve, after which the average was used for analysis. 

### 2.7. RNA Scope^®^

*Mfp2* transcripts were visualized with in situ hybridization using the RNAScope^®^ 2.5 HD Chromogenic Detection Kit (Advanced Cell Diagnostics, Newark, USA), according to the manufacturer’s protocol. In short, eyes were enucleated, fixed for 18 h in 4% PFA and embedded in paraffin, after which 7 µm thick transverse retinal sections were cut. These were subsequently hybridized with the *Mfp2* probe, followed by some amplification steps and chromogenic detection with fast red. In each experiment, a positive (directed to cyclophilin B (PPIB)) and negative (directed to Bacillus subtilis dihydrodipicolinate reductase (dapB)) control probe was included. Images were acquired with a Leica SP8× confocal microscope.

### 2.8. RPE Flatmounts 

After fixation of the enucleated eyes for 1 h in 4% PFA, the connective tissue, optic nerve, anterior segments and neural retina were removed. Next, four radial incisions were made in the remaining eyecup (RPE, choroid, sclera), after which it was blocked (10% (*v*/*v*) normal goat serum in 0.3% (*v*/*v*) Triton X-100 in PBS) and overnight incubation at 4°C with PLIN2 antibodies. Subsequently, the RPE flatmounts were probed with Alexa Fluor IgG coupled secondary antibody (1/200) and Alexa Fluor™ 647 Phalloidin antibodies overnight at 4 °C. Lastly, the RPE flatmounts were counterstained with Hoechst 33342 and mounted. Images were acquired with a Leica SP8× confocal microscope.

### 2.9. Transmission Electron Microscopy 

Preparation of eyes for transmission electron microscopy (TEM) was executed as previously described [19]. In short, mice were deeply anesthetized, followed by transcardial perfusion with Hanks’ Balanced Salt Solution (HBSS, Gibco, Bleiswijk, The Netherlands) with heparin (5 U/mL, Leo Pharma, Lier, Belgium). Subsequently, transcardial perfusion with fixative solution (2.5% (*v*/*v*) glutaraldehyde in 0.05 M sodium cacodylate (pH 7.3)) was executed. Next, eyes were enucleated, and cornea and lens were removed, followed by overnight fixation at 4 °C, using the same fixative solution. Further processing of the eyecups was performed as previously described [37]. Transmission electron images were taken with a JEOL JEM1400 microscope (JEOL Europe BV, Nieuw-Vennep, The Netherlands) (VIB Bio Imaging Core, Leuven Platform).

### 2.10. Statistics 

At least four animals were used for each experiment, with a maximum of eight. Statistical analysis was performed using the GraphPad Prism software (version 9.3). Grubbs test was executed on every data set to identify possible outliers, Shapiro–Wilk test was used to assess normal distribution and the F-test was performed to test equality of the variances. Unpaired *t*-test was performed for PR layer thickness measurements, Western blot data, PKCα positive bipolar cell count and spatial frequency, whereas two-way ANOVA was used to analyze the (flicker) ERG responses. Lipidome results (original values) were analyzed with the unpaired *t*-test, unless the diverging variability of control and mutant samples required a Welch’s *t*-test. Statistical significance was set at *p* < 0.05 and data are presented as mean ± SD.

## 3. Results

### 3.1. Confirmation of MFP2 Deletion in Crx-Mfp2^−/−^ Mice

**Figure 1:**
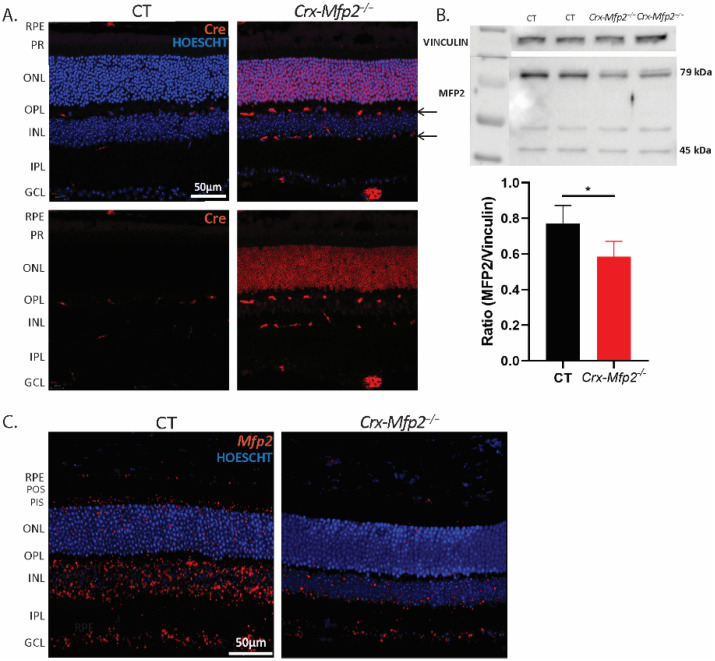
Confirmation of conditional *Mfp2* knockout in *Crx-Mfp2*^−/−^ mice. (**A**) Immunohistochemical staining for Cre recombinase (red) showed no specific staining in control mice, but clear expression in photoreceptors. Additionally, some expression was seen in the INL in *Crx-Mfp2*^−/−^ mice (arrows). (**B**) Western blotting confirmed loss of MFP2 in the neural retina of *Crx-Mfp2*^−/−^ mice. The full length MFP2 band and the hydratase band were combined to calculate MFP2 levels. The band around 50 kDa is believed to be aspecific. Vinculin was used as loading control. (**C**) In situ hybridization showed substantial loss of *Mfp2* transcripts (red) in the PR, PIS and INL of *Crx-Mfp2*^−/−^ mice. *N* = 4/group. Statistical difference based on unpaired *t*-test: * *p* < 0.05. Error bars indicate SD. RPE—retinal pigment epithelium; PR—photoreceptor layer; POS—photoreceptor outer segments; PIS—photoreceptor inner segments; ONL—outer nuclear layer; OPL—outer plexiform layer; INL—inner nuclear layer; IPL—inner plexiform layer; GCL—ganglion cell layer; MFP2—multifunctional protein 2.

To validate the activity of Cre recombinase under the control of the *Crx* promotor in *Crx-Mfp2^−/−^* mice, first immunohistochemical staining for Cre was performed (Figure 1A). This revealed ubiquitous Cre expression in the photoreceptor nuclei of *Crx-Mfp2*^−/−^ mice, while no such staining was observed in the control mice. In addition, some staining was seen in interneuron cells, which most likely represents the bipolar cells, as it has been shown before that the *Crx* promotor is active in these cells as well [25]. Next, loss of MFP2 protein in the neural retina was confirmed via Western blotting. MFP2 is a 79 kDa protein, which, upon entering the peroxisomes, is processed into a 35 kDa fragment, corresponding to the dehydrogenase domain, and a 45 kDa fragment, corresponding to the hydratase domain [8]. Using an antibody that recognized the full length MFP2 and the hydratase domain, a significant reduction in MFP2 protein levels was observed in the neural retina of *Crx-Mfp2*^−/−^ mice (Figure 1B and Figure S1). As expected, MFP2 was not completely deleted, due to the presence of untargeted interneuron cell types in the neural retina [25]. 

To visualize where MFP2 was deleted in the neural retina, in situ hybridization for *Mfp2* transcripts was performed (Figure 1C). In agreement with the Cre staining, a clear loss of *Mfp2* mRNA was seen in the photoreceptors as well as in the INL, pointing to the loss of *Mfp2* in bipolar cells. 

### 3.2. Normal Retinal Morphology and Photoreceptor Length in Crx-Mfp2^−/−^ Mice

**Figure 2:**
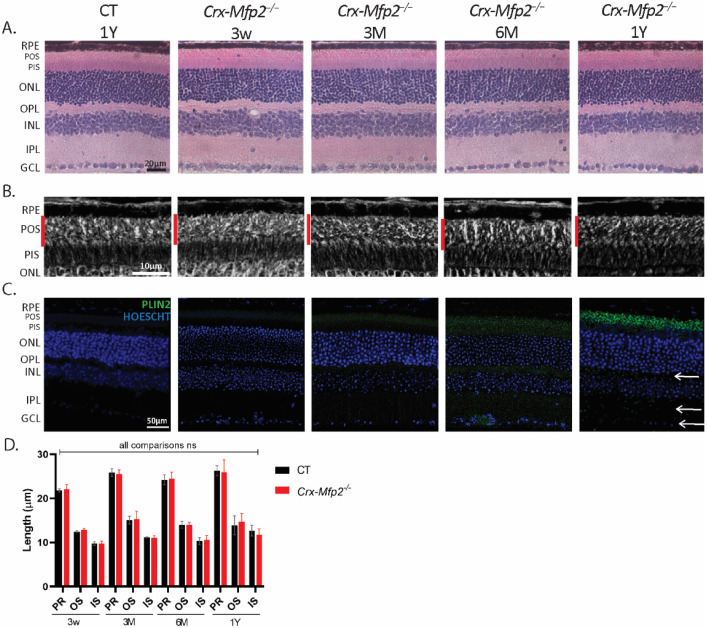
The morphology of the retina is normal in *Crx-Mfp2^−/−^* mice. (**A**) HE staining did not reveal morphological changes in *Crx-Mfp2^−/−^* mice at any investigated age. (**B**,**D**) No shortening of the photoreceptor layers is observed with phase contrast microscopy. (**C**) PLIN2 (green) staining showed a gradual increase in lipid droplet accumulation, primarily in the POS, to a lesser extent in the PIS, and sporadically in the OPL, IPL and GCL (white arrows) of *Crx-Mfp2^−/−^* mice. *N* = 4–5/group. Statistical analysis based on unpaired *t*-test of CT vs. mutant mice at each age. ns—not significant. Error bars indicate SD. RPE—retinal pigment epithelium; PR—photoreceptor layer; POS—photoreceptor outer segments; PIS—photoreceptor inner segments; ONL—outer nuclear layer; OPL—outer plexiform layer; INL—inner nuclear layer; IPL—inner plexiform layer; GCL—ganglion cell layer; PLIN2—perilipin 2.

In global *Mfp2*^−/−^ mice, POS shortening already developed at the age of 3 w and loss of photoreceptor nuclei was observed at 8 w [19]. To explore the retinal structure of *Crx-Mfp2*^−/−^ mice, HE staining was performed at the same ages, but no abnormalities were observed (Figure 2A and Figure S2 (comparison with global *Mfp2*^−/−^ mice)). Surprisingly, even at later ages until the age of 1 year, no morphological changes were noticed in the *Crx-Mfp2^−/−^* mice (Figure 2A). In addition, morphometric analysis was executed on phase contrast microscopy images, which showed no shortening of the photoreceptor layers at any age investigated (Figure 2B,D). Furthermore, rod- and cone-specific staining did not reveal any abnormalities in photoreceptor morphology of *Crx-Mfp2^−/−^* mice (Figure S3). These observations revealed that deletion of MFP2 from photoreceptors does not impact their length and survival.

Furthermore, as global *Mfp2^−/−^* mice presented with a distorted RPE in which lipids accumulated [19], the RPE health of the *Crx-Mfp2^−/−^* mice was assessed. Hereto, a double staining for phalloidin (F-actin cytoskeleton marker) and perilipin 2 (PLIN2) was performed on RPE flatmounts of 6-month-old *Crx-Mfp2^−/−^* mice. This revealed that the RPE maintained its hexagonal shape and did not accumulate lipid droplets (Figure S4).

### 3.3. Normal DHA Levels but Accumulation of VLC-PUFAs in Crx-Mfp2^−/−^ Mice

**Figure 3:**
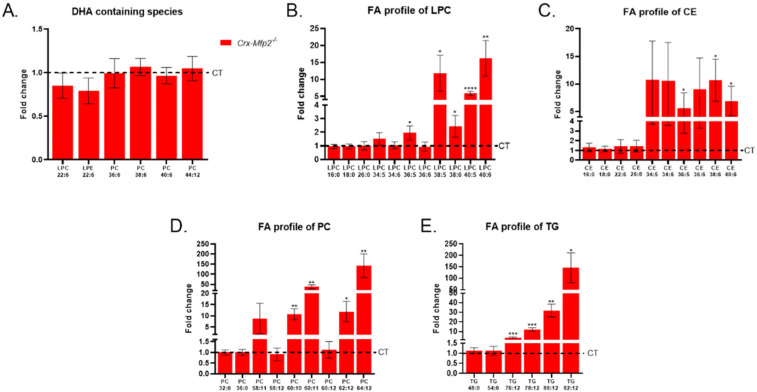
PUFA levels in the neural retina of 6-month-old *Crx-Mfp2^−/−^* mice. (**A**) DHA levels were not altered in the neural retina of *Crx-Mfp2^−/−^* mice. However, VLC-PUFAs accumulated in lysophosphatidylcholine (**B**), cholesterol esters (**C**), phosphatidylcholine (**D**) and triglycerides (**E**). Data are presented as fold change compared to CT levels. CT levels are presented as a dashed line. N = 4/group. Statistical analysis is explained in materials and methods (Section 2.10). Error bars indicate SD. DHA—docosahexaenoic acid; VLC-PUFA—very long chain polyunsaturated fatty acid; LPC—lysophosphatidylcholine; CE—cholesterol ester; PC—phosphatidylcholine; TG—triglyceride. * *p* < 0.05, ** *p* < 0.01, *** *p* < 0.001, **** *p* < 0.0001.

It was shown decades ago that local synthesis of DHA is possible in photoreceptors [21,22,23,24]. Furthermore, the global *Mfp2^−/−^* mice showed a significant reduction in DHA-containing phospholipid species as early as 3 weeks of age [19]. To investigate the potential contribution of peroxisomes in photoreceptors to DHA levels in the neural retina, lipidome analysis was performed on the neural retina of 6-month-old *Crx-Mfp2^−/−^* mice. Remarkably, the levels of lysophospholipids that contain one DHA moiety were normal in the neural retina of the *Crx-Mfp2^−/−^* mice (Figure 3A). Furthermore, phosphatidylcholine (PC) species containing most likely one DHA moiety (PC (36:6), PC (38:6), PC (40:6)) or two DHA moieties (PC (44:12)) were not altered. These findings question the contribution of local synthesis of DHA to the DHA pool in the neural retina.

The lipidomics data were further analyzed with regard to the fatty acid profile. Because lysophosphatidylcholines (LPC) and cholesterol esters (CE) contain only one fatty acid, their composition was first assessed. Interestingly, species containing VLC-PUFAs (≥C36) accumulated in the neural retina of 6-month-old *Crx-Mfp2^−/−^* mice (Figure 3B,C). Unexpectedly, the saturated VLCFA LPC (26:0) was not altered (Figure 3B). In addition, the levels of C16:0 and C18:0 fatty acids esterified in LPC and cholesterol esters were similar in control and *Crx-Mfp2^−/−^* retinas (Figure 3B,C). The normal levels of these common fatty acids was confirmed by checking PC species containing 32C (2× C16:0) and 36C (2× C18:0) and triglyceride (TG) species that contain 48C (3× C16:0) and 54C (3× C18:0) in their fatty acid residues (Figure 3D,E). In contrast, PC and TG species that contain more than 60C and 76C, respectively, and at least 6 double bonds, were increased 10–150-fold (Figure 3D,E). The branched chain fatty acids, phytanic acid and pristanic acid were not elevated in any of the lipids investigated (data not shown). Although our analysis does not allow a comparison between the absolute concentrations of different lipid species, it is generally known that VLC-PUFAs are very low in concentration compared with long chain saturated or monounsaturated fatty acids. To evaluate whether this resulted in the accumulation of neutral lipids, lipid droplet staining was performed using PLIN2 antibodies (Figure 2C). From 3 M of age, lipid droplets started to accumulate in the POS, which gradually increased over time, leading to a striking abundance of lipid droplets in the POS of 1-year-old *Crx-Mfp2^−/−^* mice. Furthermore, from 6 M of age, lipid droplets were also more numerous in the PIS, OPL, IPL and ganglion cell layer. These findings suggest that peroxisomal β-oxidation in the neural retina is not essential for the DHA pool, but rather to prevent the accumulation of VLC-PUFAs.

### 3.4. Crx-Mfp2^−/−^ Mice Present with Reduced Visual Acuity and a Negative ERG

**Figure 4:**
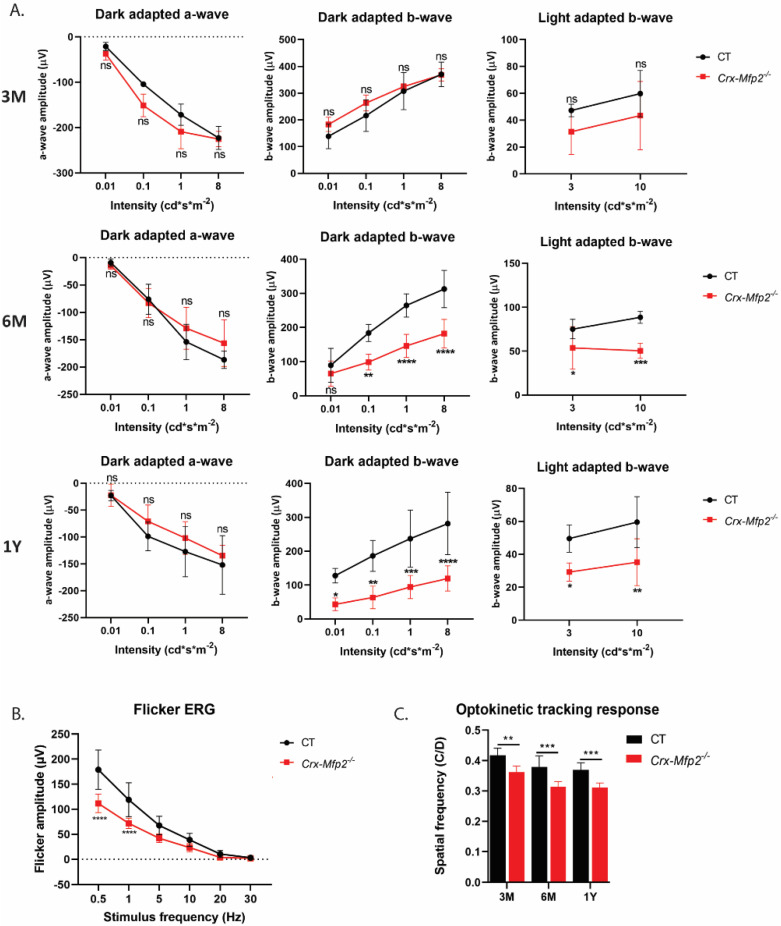
Reduced visual acuity and function in *Crx-Mfp2^−/−^* mice. (**A**) No change in scotopic a-wave between control and *Crx-Mfp2^−/−^* mice at any investigated age. The scotopic and photopic b-wave was significantly reduced in 6 M and 1 Y old *Crx-Mfp2^−/−^* mice. (**B**) Flicker ERG responses were impaired (6 M). (**C**) Optokinetic tracking response showed a significant reduction in visual acuity at 3 M, 6 M and 1 Y. N = 4–8/group. Statistical difference based on unpaired t-test and two-way ANOVA: * *p* < 0.05, ** *p* < 0.01, *** *p* < 0.001, **** *p* < 0.0001; ns—not significant. Error bars indicate SD.

Global *Mfp2^−/−^* mice already presented with reduced visual function at 3 weeks and reduced visual acuity at the age of 8 weeks [19]. To evaluate the visual function of *Crx-Mfp2^−/-^* mice, dark and light adapted full-field ERG was conducted at 3 w (data not shown), 3 M, 6 M and 1 Y. The scotopic a-wave, which represents the activity of the photoreceptors, did not show a difference between control and *Crx-Mfp2^−/−^* mice at any investigated age, which is in coherence with the observed normal photoreceptor length and number (Figure 4A). The b-wave, on the other hand, which represents the activity of the interneurons, showed a significantly reduced response in both scotopic and photopic conditions, starting from the age of 6 M and deteriorating over age (Figure 4A). A selective reduction in the b-wave is also called a negative ERG [38,39]. The b-wave originates from the depolarization of the inner retinal cell types. To identify which of these inner retinal cell types were affected, flicker ERG was performed to specifically measure bipolar cell activity. Bipolar cells connect the photoreceptors to the ganglion cells and are therefore essential to transmit the light signal. There are two different types of bipolar cell responses: ON bipolar cells depolarize in response to light stimuli and OFF bipolar cells hyperpolarize in response to light. Furthermore, there are rod- and cone-specific bipolar cells [40]. The rod bipolar cell (ON) pathway is measured below 5 Hz, the cone ON pathway between 5 and 15 Hz and the cone OFF pathway above 15 Hz [33,34]. Intriguingly, in *Crx-Mfp2^−/−^* mice the rod bipolar pathway is significantly reduced, and the cone bipolar pathway showed a tendency to reduction (Figure 4B). To further evaluate whether this affects the visual acuity, the optokinetic tracking response was measured at 3 M, 6 M and 1 Y of age. This revealed a significant reduction in visual acuity in the *Crx-Mfp2^−/−^* mice (Figure 4C).

### 3.5. Crx-Mfp2^−/−^ Mice Exhibit Impaired Synaptic Integrity and Loss of Bipolar Cells

**Figure 5:**
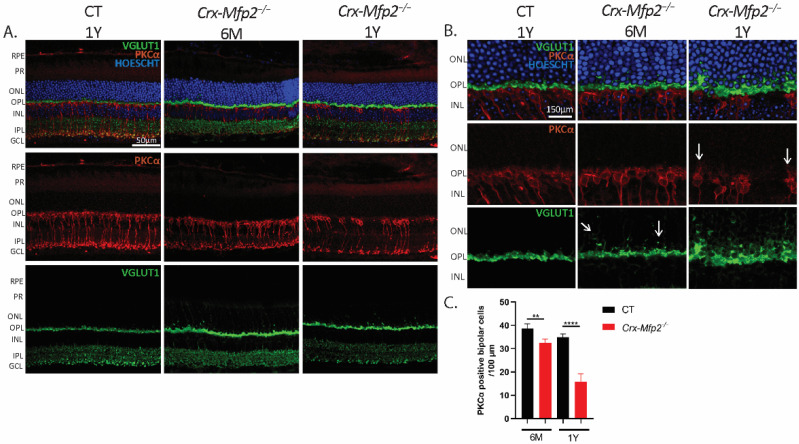
*Crx-Mfp2^−/−^* mice show loss of bipolar cells and impaired synaptic connections. (**A**) PKCα (red) staining showed clear loss of bipolar cells at the age of 1 Y. (**B**) Higher magnification images revealed sprouting of bipolar cells (red) and VGLUT1 (green) mislocalization into the ONL. White arrows indicate the sprouting. (**C**) Quantification of PKCα positive bipolar cells per 100 µm revealed a significant loss in both 6-month-old and 1-year-old *Crx-Mfp2^−/−^* mice. N = 4/group. Statistical difference based on unpaired *t*-test: ** *p* < 0.01, **** *p* < 0.0001. Error bars indicate SD. RPE—retinal pigment epithelium; PR—photoreceptor; ONL—outer nuclear layer; OPL—outer plexiform layer; INL—inner nuclear layer; IPL—inner plexiform layer; GCL—ganglion cell layer.

As the flicker ERG findings indicated that the rod bipolar cell pathway is impaired in *Crx-Mfp2^−/−^* mice, the morphology of the bipolar cells was investigated using IHC staining for the rod bipolar cell specific marker PKCα. Interestingly, the *Crx-Mfp2^−/−^* mice showed a slight reduction in PKCα positive bipolar cells at 6 M and a remarkable loss of bipolar cells at the age of 1 Y (Figure 5A,C). Next, the synaptic connections between the photoreceptors and the bipolar cells were investigated. Hereto, presynaptic photoreceptor terminals were stained with VGLUT1 and the bipolar cell synapses with PKCα. Strikingly, VGLUT1 mislocalized into the ONL already at 6 M and bipolar cells sprouted into the ONL at the age of 1 Y (Figure 5B). Interestingly, double staining of PLIN2 with PKCα, revealed that lipid droplets accumulated in the rod bipolar cells (Figure S5). 

**Figure 6:**
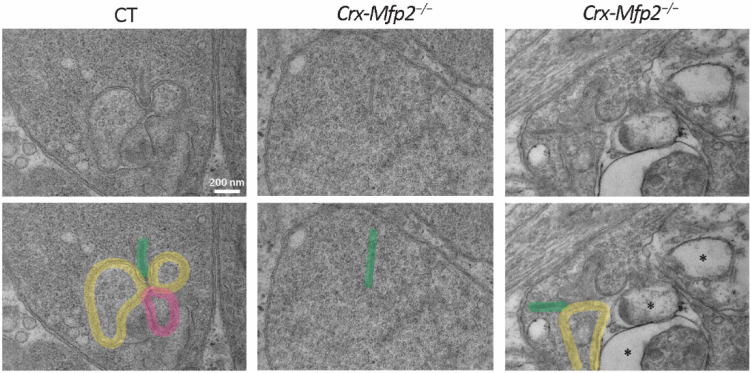
Loss of photoreceptor ribbon synapse integrity in 6-month-old *Crx-Mfp2^−/−^* mice. Transmission electron microscopy images showed normal photoreceptor ribbon synapse formation in CT mice, with a ribbon (green), two horizontal cells (yellow) and a bipolar cell (pink). *Crx-Mfp2^−/−^* mice presented with free-floating photoreceptor ribbons and abnormal structures (*) in the photoreceptor synapses, which did not occur in CT mice. Top and bottom panel are the same images. Bottom panel includes interpretation. N = 4/group.

To have a closer look at the synaptic connections between the photoreceptors and the inner retinal cell types, transmission electron microscopy was performed on 6-month-old *Crx-Mfp2^−/−^* mice. The photoreceptor synaptic terminal consists of a specialized synaptic ribbon which is an electron dense structure that is attached to the membrane and projects vertically in the cytoplasm of the synapse. It has a function in the tethering and trafficking of glutamate containing vesicles. This synaptic ribbon makes contact with invaginating horizontal and bipolar cells [41]. Strikingly, synaptic ribbons were floating freely in the cytoplasm of the photoreceptor synaptic terminal of *Crx-Mfp2^−/−^* mice, suggesting a loss of connection between the photoreceptor ribbon synapse and the post-synaptic terminals from the inner retinal cell types (Figure 6 and Figure S6). Entirely free-floating ribbon synapses were occasionally present, while ribbon synapses connected to one horizontal cell were predominantly observed. Some normally formed ribbon-synapses were detected as well. Furthermore, abnormal vacuoles were seen, and the presynaptic vesicles seemed to be less dense and bigger in size in the *Crx-Mfp2^−/−^* mice (Figure 6). Taken together, loss of MFP2 gives rise to impaired integrity of the photoreceptor ribbon synapse, thereby harming the synaptic connection and thus the synaptic transmission between the photoreceptors and the bipolar cells.

### 3.6. Increased Inflammation in Crx-Mfp2^−/−^ Mice 

**Figure 7:**
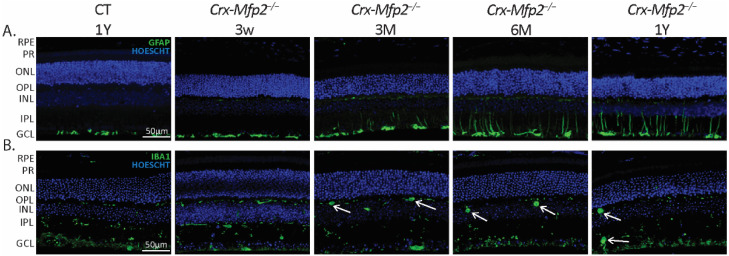
Retinal inflammation in the *Crx-Mfp2^−/−^* mice. (**A**) Activated Müller cells were observed starting from the age of 3 M. Progressive sprouting into the retinal layers is observed at later time points. (**B**) Microglia infiltration is seen in the OPL already from 3 M of age. From 6 M microglia are swollen and reactive both in the OPL and IPL (white arrows). N = 4/group. RPE—retinal pigment epithelium; PR—photoreceptor layer; ONL—outer nuclear layer; OPL—outer plexiform layer; INL—inner nuclear layer; IPL—inner plexiform layer; GCL—ganglion cell layer; GFAP—glial fibrillary acidic protein; IBA1—ionized calcium binding adaptor molecule 1.

Inflammation is a common feature in numerous retinal pathologies. The universal retinal response to stress is the increased expression of glial fibrillary acidic protein (GFAP) in Müller cells, and microglia infiltration in the retina [42,43]. To investigate a possible retinal stress response in the retina of *Crx-Mfp2^−/−^* mice, IHC staining for GFAP was performed, revealing activated Müller cells starting from 3 M of age (Figure 7A). Furthermore, the distribution and size of microglia was evaluated, using the microglia marker ionized calcium binding adaptor molecule 1 (IBA1). Microglia normally reside in the OPL and IPL and display elongated dendrites, but when activated become rounded and infiltrate into the retinal layers. Remarkably, the *Crx-Mfp2^−/−^* mice showed activated microglia in the OPL and IPL already at 3 M which progressed over time (Figure 7B). As both the Müller glia and microglia have the wildtype genotype, their reactivity is a consequence of the abnormalities in the photoreceptors and/or bipolar cells. Furthermore, it can be concluded that the retinal stress response is activated in *Crx-Mfp2^−/−^* mice already at an early stage.

To investigate whether there is oxidative stress along with the inflammation and lipid accumulations in the retina of *Crx-Mfp2^−/−^* mice, staining for 4-HNE, a product of lipid peroxidation, was performed at 1 Y of age. No obvious increase in 4-HNE staining was observed (Figure S7), suggesting that loss of peroxisomal β-oxidation in the photoreceptors and bipolar cells does not result in oxidative stress. 

### 3.7. Crx-Pex5^−/−^ Mice Do Not Present with a More Severe Retinal Phenotype than Crx-Mfp2^−/−^ Mice

**Figure 8:**
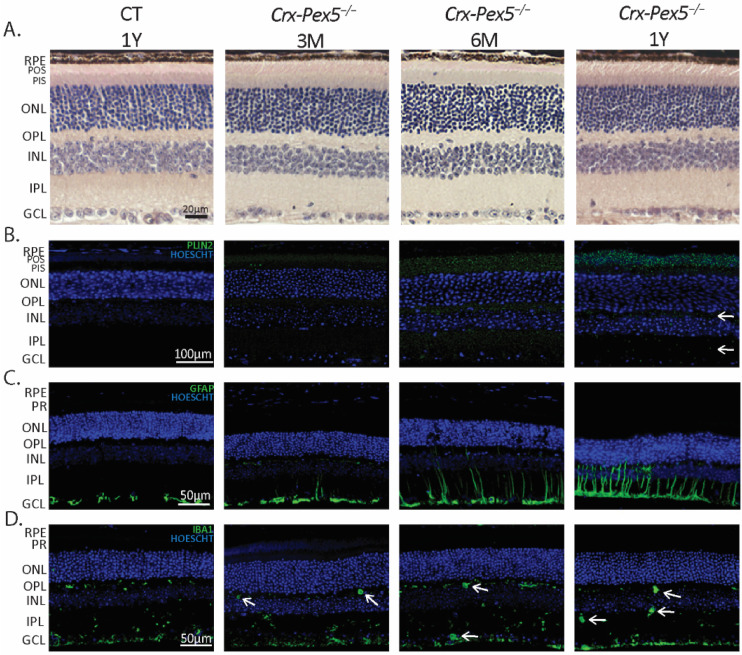
Retinal phenotype of *Crx-Pex5^−/−^* mice. (**A**) HE staining showed no morphological differences between CT and *Crx-Pex5^−/−^* mice at any investigated age. (**B**) Lipid droplet staining revealed a gradual increase in lipid droplets in the POS, PIS, OPL (white arrows), IPL (white arrows) and GCL in *Crx-Pex5^−/−^* mice. (**C**) Activated Müller cells were observed starting from the age of 3 M, with progressive sprouting into the neural retina at later time points. (**D**) Microglia are swollen and reactive both in the OPL and IPL (white arrows) from 3 M of age. N = 4/group. RPE—retinal pigment epithelium; PR—photoreceptor; POS—photoreceptor outer segments; PIS—photoreceptor inner segments; ONL—outer nuclear layer; OPL—outer plexiform layer; INL—inner nuclear layer; IPL—inner plexiform layer; GCL—ganglion cell layer; GFAP—glial fibrillary acid protein; IBA1—ionized calcium binding adaptor molecule 1; PLIN2—perilipin 2.

The mild phenotype of *Crx-Mfp2^−/−^* mice at the level of photoreceptors was unexpected in view of the abundance of peroxisomes in the inner segments of photoreceptors. This raised the question whether peroxisomes might be essential in photoreceptors for activities not related to β-oxidation. This was investigated by generating mice with a conditional deletion of *Pex5* (*Crx-Pex5^−/−^*) using the same *Crx-Cre* mice. After confirmation of PEX5 knockout by assessing its function (Figure S8), the retinal morphology was investigated (Figure 8A). Interestingly, no structural changes were observed in the retina of *Crx-Pex5^−/−^* mice until the age of 1 year. Therefore, it can be concluded that peroxisomes localized in the photoreceptors are not important for their number and POS length. 

**Figure 9:**
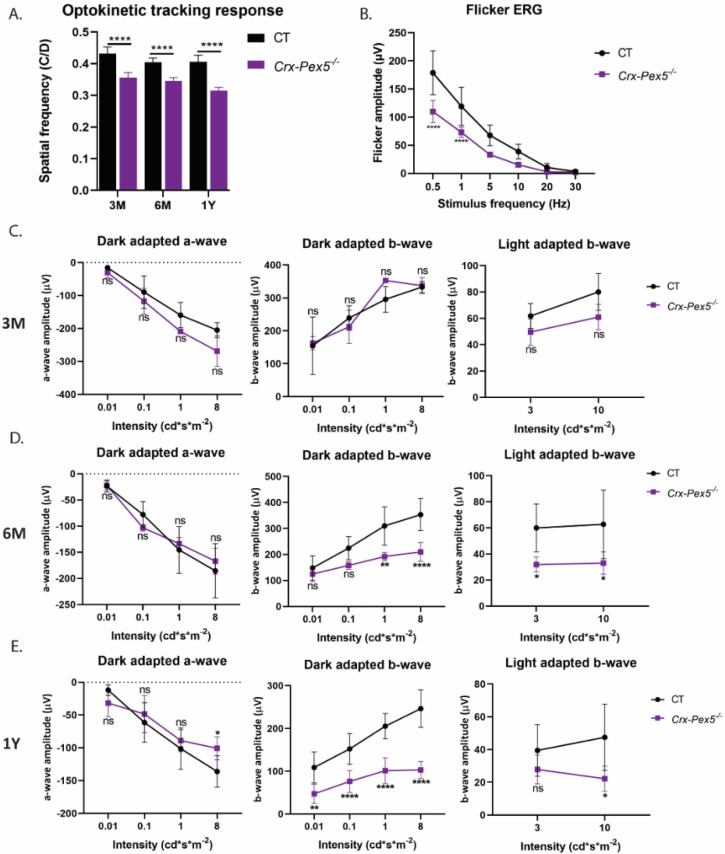
Reduced visual acuity and function in *Crx-Pex5^−/−^* mice. (**A**) Optokinetic tracking response showed a significant reduction in visual acuity starting at 3 M. (**B**) Flicker ERG responses are lower in 6-month-old *Crx-Pex5^−/−^* mice. (**C**–**E**) Scotopic and photopic b-wave responses are significantly affected in *Crx-Pex5^−/−^* mice starting from 6 M of age. N = 4/group. Statistical difference based on two-way ANOVA and unpaired *t*-test: * *p* < 0.05, ** *p* < 0.01, **** *p* < 0.0001; ns—not significant. Error bars indicate SD.

The functional consequences of peroxisome inactivation were then investigated by assessing the optokinetic tracking response, full-field and flicker ERG of the *Crx-Pex5^−/−^* mice. Again, a reduced visual acuity was observed at all investigated ages (Figure 9A). This was not related to impaired photoreceptor function as the dark- and light-adapted, full-field ERG showed no significant reduction in the a-wave at any age, except at the highest intensity in 1-year-old *Crx-Pex5^−/−^* mice (Figure 9C–E). Interestingly, the blunted b-wave response and flicker ERG amplitude in the *Crx-Pex5^−/−^* mice was similar compared to the *Crx-Mfp2^−/−^* mice (Figure 9B–E). Moreover, staining for PKCα and VGLUT1 revealed the same phenotype as the *Crx-Mfp2^−/−^* mice (Figure S9). There was mislocalization of VGLUT1 into the ONL already at 6 M and loss of bipolar cells at 1 Y of age. Analogous to *Crx-Mfp2^−/−^* mice, these alterations in the neural retina were accompanied by accumulation of lipid droplets and extensive activation of Müller glia and microglia in the retina of the *Crx-Pex5^−/−^* mice (Figure 8B–D). 

Taken together, the similarity in phenotype of *Crx-Mfp2^−/−^* and *Crx-Pex5^−/−^* mice suggests that peroxisomal β-oxidation is the most important function of peroxisomes in photoreceptors/bipolar cells.

## 4. Discussion

Previously, we showed that intact peroxisomal β-oxidation is of crucial importance for retinal integrity, as global *Mfp2^−/−^* mice presented with a complex and severe retinal phenotype [19]. The underlying mechanism, however, was still to be determined. Several lines of evidence pointed to a role of peroxisomal β-oxidation in photoreceptors, which was investigated here using *Crx-Mfp2^−/−^* and *Crx-Pex5^−/−^* mice. Surprisingly, impaired peroxisomal β-oxidation, and even loss of functional peroxisomes in photoreceptors, did not affect their length, number or phototransduction process, but their synaptic terminals were disorganized. In contrast, bipolar cells which were also targeted, showed impaired functioning and survival. 

### 4.1. Peroxisomal β-Oxidation in Photoreceptors Is Not Essential for Their Survival, Function and DHA Content

Global *Mfp2^−/−^* mice develop an early onset retinal degeneration, including reduced POS length and photoreceptor cell death already at 3 w, accompanied by almost complete absence of DHA containing phospholipid species [19]. As in situ hybridization for *Mfp2* mRNA showed high expression of *Mfp2* in the photoreceptor inner segments [19] and local synthesis of DHA is possible in photoreceptors [21,22,23,24], we hypothesized that peroxisomes in the photoreceptors could play a role in this retinal phenotype. Surprisingly, both *Crx-Mfp2^−/−^* and *Crx-Pex5^−/−^* mice retained normal POS length, photoreceptor numbers and ERG a-waves until 1 Y of age. These findings suggest that the early photoreceptor death in global *Mfp2^−/−^* mice is not driven cell autonomously and it raises the question whether rather the RPE cells play an important role. Therefore, it would be interesting to investigate the consequences of RPE specific deletion of *Mfp2* on the retina. 

In addition, lipidome analysis on the neural retina of *Crx-Mfp2^−/−^* mice showed normal levels of DHA containing phospholipid species. These findings demonstrate that local synthesis of DHA in the neural retina is only of minor importance compared with the systemic supply. Furthermore, it indicates that the reduced DHA levels in the global *Mfp2^−/−^* mice might be of great significance for the observed retinal phenotype. To confirm the necessity of the systemic supply for the DHA pool in the neural retina, it would be informative to investigate the retinal phenotype of liver specific *Pex5* knockout mice (*Alb-Pex5^−/−^*) [44].

### 4.2. Peroxisomal β-Oxidation Is Crucial for VLC-PUFA Homeostasis That May Affect the Synapse

Lipidome analysis on the neural retina of global *Mfp2^−/−^* mice revealed a peculiar profile of PC species containing one DHA moiety and one VLC-PUFA moiety. Phospholipid species containing up to 56 carbons were severely decreased, while those containing more than 56 carbons were unchanged or elevated [19]. Interestingly, the *Crx-Mfp2^−/−^* mice showed a similar phenotype: levels of phospholipid species containing up to 56 carbons were normal, while those above 56 carbons accumulated. Furthermore, VLC-PUFAs accumulated in cholesterol esters and triglycerides. These findings show that peroxisomal β-oxidation in the neural retina is essential to break down VLC-PUFAs. 

The MFP2 mouse models can be considered the counterpart of pathologies with a lack of VLC-PUFA levels. The latter occurs in patients with autosomal dominant Stargardt-3 macular dystrophy (STGD3), which is caused by a mutation in the elongation of very long chain fatty acids 4 (*Elovl4)* gene [45]. This enzyme is responsible for the elongation of lipids containing more than 26 carbons. While contradictory results were obtained in the different mouse models, the most recent model with a conditional deletion of *Elovl4* from both rods and cones (*Chx10-Elovl4^−/−^*), showed barely detectable levels of VLC-PUFAs in the retina, resulting in a negative ERG [46,47]. Bennett et al. showed in this model that VLC-PUFAs are not only highly expressed in the POS, but also in the photoreceptor ribbon synapses [47]. The exact role of VLC-PUFAs in these retinal synapses is not completely clear yet. It was hypothesized that the van der Waals interactions between the long lipid chains are responsible for the stabilization of the synaptic vesicle bilayer, thereby hindering membrane fusion and content release [13,48]. On the other hand, the degree of unsaturation in these long lipid chains can influence the fluidity and therefore destabilize the vesicle membranes [13,48]. 

An additional abnormality in photoreceptor synapses of the *Chx10-Elovl4^−/−^* mouse is the smaller size of synaptic vesicles [47]. Remarkably, TEM images of the synapses of *Crx-Mfp2^−/−^* mice revealed larger and less dense synaptic vesicles. It thus seems that both a depletion and an excess of VLC-PUFAs affect synaptic vesicle dynamics and function. 

### 4.3. Bipolar Cell Survival and Functioning Depend on Peroxisomal β-Oxidation

The negative ERG, i.e., impaired b-waves despite normal a-waves, in *Crx-Mfp2^−/−^* and *Crx-Pex5^−/−^* mice also points to a hampered signal transduction at the level of photoreceptor–bipolar cells. In other mouse models negative ERGs were related to either presynaptic defects in photoreceptor terminals or postsynaptic defects in bipolar cells [38,39,41]. Presynaptic defects result for example from incorrect assembly or functioning of the photoreceptor ribbon synapse, such as in *Bsn^Ex4/5^*, *Ribeye^−/−^* and *Crx-Pomt1^−/−^* mutant mice [49,50,51,52]. Typically, free-floating ribbons occur in the photoreceptor synaptic terminal, but bipolar cells are preserved. Postsynaptic impairment is seen in mouse models with loss of genes important for the depolarization of bipolar cells (*mGluR6^−/−^* [53,54] and *Gβ5^−/−^* [55]) or bipolar cell specific deletion of genes either required for insertion of membrane proteins into the ER membranes (*Pcp2-Emc3^−/−^* [56]) or responsible for the proper functioning of flippases that regulate the distribution of phospholipids in the membrane bilayer (*Pcp2-Tmem30^−/−^* [57]). Only this last model has any relation with lipids as the cause of a negative ERG. In most of these postsynaptic mutant mice, bipolar cells degenerate and die, but the photoreceptor ribbon synapse is never affected. 

A common theme in all these mouse models is ectopic sprouting of photoreceptor–bipolar cell synapses. Whereas these synapses are normally confined to the OPL, extended aberrant processes into the ONL are seen. Likewise, both the *Crx-Mfp2^−/−^* and *Crx-Pex5^−/−^* mice showed a remarkable degree of sprouting into the ONL, visualized by the ectopic location of VGLUT1 and PKCα starting from 6 M and 1 Y of age, respectively. Considering that free-floating ribbon synapses as well as bipolar cell death occur in the peroxisome deficient models, we speculate that both photoreceptor synaptic terminals and bipolar cells are damaged due to the impaired peroxisomal β-oxidation. It remains to be determined whether these are independent phenomena or whether defective photoreceptor terminals affect bipolar cells or vice versa. The mislocalization and loss of integrity of the photoreceptor ribbon synapses already at 6 M of age, followed by mislocalization and loss of bipolar cells at 1 Y of age, suggests that the observed bipolar cell defects might be secondary to the photoreceptor synaptic issues. This can be confirmed by studying the retinal phenotype of bipolar cell specific *Mfp2* knockouts. 

### 4.4. Retinal Gene Augmentation for Peroxisomal Disorders Should Target the Entire Retina 

Over the years, several mouse models for peroxisomal disorders have been developed [58]. However, until now only the retinal phenotype of the *Mfp2^−/−^* mouse and the *Pex1* knockin mouse, homozygous for the most common human *PEX1* mutation (G843D) (*Pex1^G844D^*), have been reported [17,19,59]. The *Pex1^G844D^* mouse presented with decreased visual acuity and function, together with abnormal cone morphology already early on [17]. Interestingly, the bipolar cells sprouted into the ONL starting from 6 M of age and subsequently died, similar to the *Crx-Mfp2^−/−^* and *Crx-Pex5^−/−^* mice [17]. Furthermore, lipidome analysis showed accumulation of the peroxisomal marker LPC (26:0). Unfortunately, VLC-PUFA levels were not reported. 

The same research group recently evaluated the effect of retinal gene augmentation therapy to either prevent or recover the retinal degeneration in the *Pex1^G844D^* mouse [20]. Until 25 weeks of age, this yielded a 2-fold increase in the ERG responses compared with non-injected *Pex1^G844D^* eyes. However, ERG amplitudes remained critically low compared to wildtype levels. Furthermore, the visual acuity did not significantly improve. Remarkably, retinal gene augmentation did reduce the LPC (26:0) levels, although they were still doubled compared with wildtype levels. Importantly, the AAV2/8 vector that was used mainly targets photoreceptor cells. As our data show that peroxisomes in the photoreceptors are not essential for their length, number and phototransduction process, targeting the whole retina might be essential to rescue the retinal phenotype. 

### 4.5. What Is the Role of Peroxisomes in the Photoreceptors? 

The absence of phenotype in the outer retina of *Crx-Mfp2^−/−^* and *Crx-Pex5^−/−^* mice was surprising and raises questions with regard to the function of peroxisomes that are abundantly present in the inner segments, as shown with staining for multiple PEX proteins [14,15,16,17,18]. As already mentioned, peroxisomes in photoreceptors are not essential for the DHA pool in the neural retina. It may be that peroxisomes are superfluous in the photoreceptors and that surrounding cell layers can take over their functions. However, the accumulation of VLC-PUFAs in phospholipids, cholesterol esters and triglycerides in the neural retina of *Crx-Mfp2^−/−^* mice suggests otherwise. It indicates that the elongation of PUFAs by ELOVL4 in the inner segments needs to be counteracted by peroxisomal β-oxidation and we hypothesize that this is the primary role of peroxisomes in photoreceptors. In contrast, LPC (26:0) and branched chain fatty acids did not accumulate in *Crx-Mfp2^−/−^* mice. This deviates from global *Mfp2^−/−^* and *Pex1* knockin mice in which LPC (26:0) levels were shown to be increased in the retina. Although we do not have an explanation for the normal LPC (26:0) levels, it further emphasizes that the imbalance of VLC-PUFAs are likely causative for the pathology. An additional observation was the gradual increase in lipid droplets, predominantly in the outer segments, but also in the inner retina. In view of the normal levels of common C16:0 and C18:0 containing lipids, we assume that these lipid reserves are primarily composed of VLC-PUFAs. It is quite striking that the vast accumulation of lipid droplets in the POS did not affect the phototransduction process (as shown by normal a-waves in ERG analysis). In contrast, the photoreceptor ribbon synapse, where VLC-PUFAs have been shown to play important roles [13,47,48], is disorganized, including free-floating ribbons and vesicles with altered size and density. To further explore the links between VLC-PUFAs and lipid droplet accumulation, it would be very informative to apply imaging MALDI-MS on the neural retina of *Crx-Mfp2^−/−^* mice. The importance of peroxisomes for the integrity of the photoreceptor synaptic terminal and synaptic vesicle dynamics raises the question if the synaptic efficacy in the brain is also affected and whether this contributes to the extensive neurological pathology of the *Mfp2^−/−^* mice. 

The lipid abnormalities were not accompanied by enhanced levels of ROS, which is in line with observations in other tissues of MFP2-deficient mice (M. Baes, unpublished observations). However, early on, both Müller glia and microglia displayed a reactive phenotype which must be due to environmental changes given that they are wildtype. It has to be taken into account that prolonged activation of these cells can induce an exaggerated pro-inflammatory phenotype, which can worsen the pathology initiated by the inactive peroxisomal β-oxidation in photoreceptors and bipolar cells [60].

## 5. Conclusions

Our data prove that the early retinal phenotype observed in the global *Mfp2^−/−^* mice cannot be explained by the lack of peroxisomal β-oxidation in photoreceptors. These findings also show that only targeting the photoreceptors in a therapeutic approach might not be sufficient to alleviate the retinal phenotype of global *Mfp2^−/−^* mice. A vital finding was that the contribution of peroxisomal β-oxidation in photoreceptors for the acquisition of DHA is only minor compared to the systemic supply. However, it is essential to break down the VLC-PUFAs that are located both in the synapse and in the discs. Furthermore, we show that peroxisomal β-oxidation is crucial for the integrity of the photoreceptor synaptic ribbon and for the maintenance and functioning of bipolar cells in adulthood. To decipher whether the issue lies presynaptic or postsynaptic, or a combination of both, further investigation in bipolar cell specific knockout mice of *Mfp2* is required. 

## Figures and Tables

**Table 1:** List of primers used for PCR genotyping.

Gene	Forward	Reverse
*Cre recombinase*	5′ GCCTGCATTACCGGTCGATGCAACGA 3′	5′ GTGGCAGATGGCGCGGCAACACCATT 3′
*Mfp2^L/L^*	5′ CCCAACGCTGGGTCACGGATGACG 3′	5′ GCAACCATAAGTTACACAAAATGCC 3′
*Pex5^L/L^*	5′ CTCTGGTTCCCATGGCCAGGGTGG 3′	5′ GGGGAGTACGACAAGGCTGTGGACTG 3′

**Table 2:** List of used antibodies for immunohistochemical staining and Western blotting.

Primary Antibody	Host	Dilution		Supplier/Reference
Cre recombinase	Mouse	1/500	Frozen	Euromedex (CRE-2D8-As)
GFAP	Rabbit	1/10,000	NDF	Dako (Z0334)
4-HNE	Rabbit	1/100	1% PFA	Calbiochem (393207)
MFP2	Rabbit	1/200	Western blotting	Proteintech (15116-1-AP)
Opsin	Rabbit	1/100	NDF	Millipore (AB5405)
Peanut agglutinin	/	1/100	NDF	Vector Laboratories (FL-1071)
Phalloidin-Alexa647	/	1/100	RPE flatmount	Invitrogen (A30107)
PKCα	Mouse	1/100	1% PFA	Santa Cruz (SC-17769)
PLIN2	Rabbit	1/1000 1/200	NDF RPE flatmount	NOVUS (NB110-40877)
Rhodopsin	Mouse	1/1000 (Alexa 1/750)	NDF	Millipore (MAB5356)
Thiolase	Rabbit	1/500	Western blotting	Van Veldhoven P.P. [35]
VGLUT1	Rabbit	1/1000	1% PFA	Synaptic Systems (135303)
Vinculin	Mouse	1/2000	Western blotting	Sigma (V9131)

GFAP—glial fibrillary protein; 4-HNE—4-hydroxy-2-nonenal; MFP2—multifunctional protein 2; PKCα—protein kinase Cα; PLIN2—perilipin 2; VGLUT1—vesicular glutamate transporter 1.

## Supplementary Materials

The following supporting information can be downloaded at: https://www.mdpi.com/article/10.3390/cells11010161/s1, Figure S1: Full blot of the confirmation of conditional *Mfp2* knockout in *Crx-Mfp2^−/−^* mice, Figure S2: Comparison of the retinal morphology of 3- and 8-week-old global *Mfp2^−/−^* and *Crx-Mfp2^−/−^* mice, Figure S3: Photoreceptor morphology is not affected in 1-year-old *Crx-Mfp2^−/−^* mice, Figure S4: RPE health is not affected in 6-month-old *Crx-Mfp2^−/−^* mice. Figure S5: Lipid droplets accumulate in the rod bipolar cells of *Crx-Mfp2^−/−^* mice, Figure S6: Additional TEM images of the photoreceptor ribbon synapse of 6-month-old *Crx-Mfp2^−/−^* mice, Figure S7: No increase in oxidative stress in the neural retina of *Crx-Mfp2^−/−^* mice, Figure S8: Full blot of the confirmation of *Crx-Pex5* knockout, Figure S9: PKCα and VGLUT1 double staining in *Crx-Pex5^−/−^* mice reveals loss of bipolar cells and ectopic sprouting into the ONL.
