# Supplementary material for: Cell Type-Selective Loss of Peroxisomal β-Oxidation Impairs Bipolar Cell but Not Photoreceptor Survival in the Retina
Source: Cells. 2022 Jan 4;11(1):161. doi: 10.3390/cells11010161 (PMC8750404; doi:10.3390/cells11010161)
Supplement: Supplementary file 1 [file cells-11-00161-s001.zip › cells-1463434-supplementary.pdf]

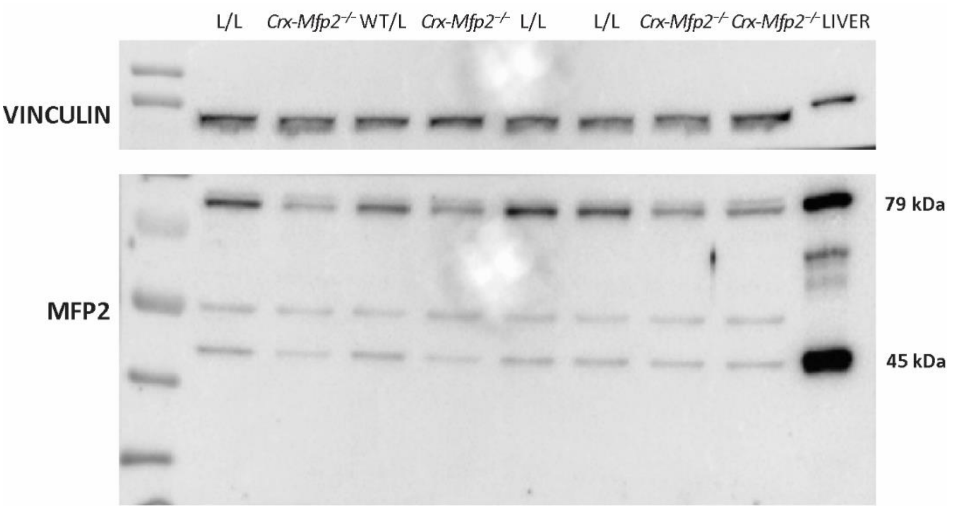

Figure S.1. *Full blot of the confirmation of conditional MFP2 knock-out in Crx-Mfp2$^{-/-}$ mice. The most right lane represents a liver sample of a wild-type mouse, used as control for the height of the MFP2 bands. Vinculin was used as loading control. MFP2: multifunctional protein 2.*

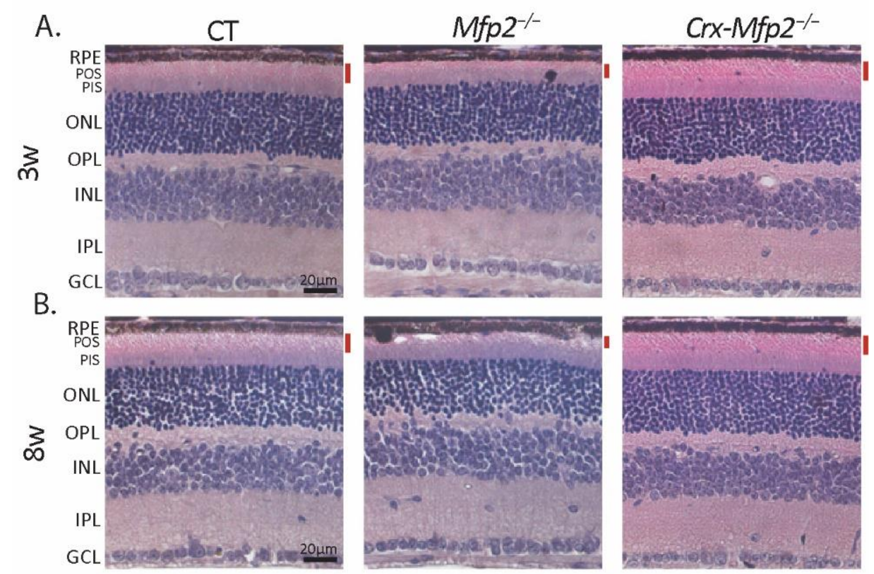

Figure S.2. *Comparison of the retinal morphology of 3- and 8-week-old global Mfp2$^{-/-}$ and Crx-Mfp2$^{-/-}$ mice.* ***A)*** *Global Mfp2$^{-/-}$ mice already presented with POS shortening at 3 weeks of age, while Crx-Mfp2$^{-/-}$ mice retained normal POS length at this age.* ***B)*** *8-week-old global Mfp2$^{-/-}$ mice presented with severe POS shortening together with mislocalization and loss of photoreceptor nuclei, which was not observed in the Crx-Mfp2$^{-/-}$ mice at the same age. RPE: retinal pigment epithelium, POS: photoreceptor outer segment, PIS: photoreceptor inner segment, ONL: outer nuclear layer, OPL: outer plexiform layer, INL: inner nuclear layer, IPL: inner plexiform layer, GC: ganglion cell layer.*

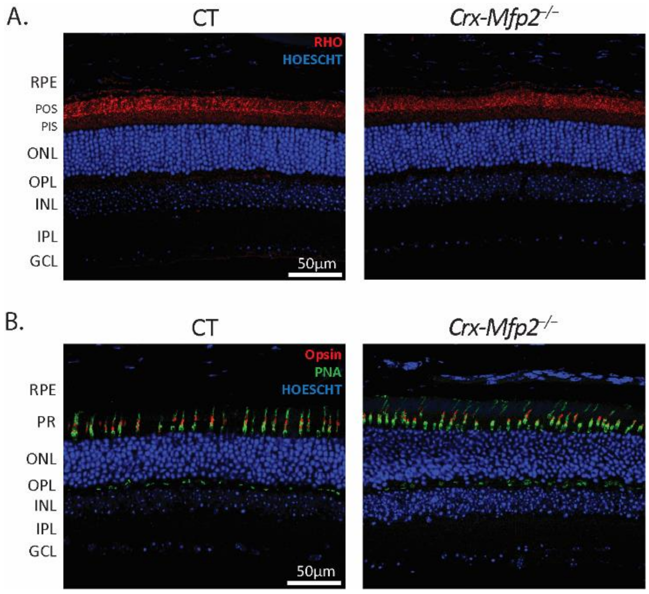

Figure S.3. *Photoreceptor morphology is not affected in 1-year-old Crx-Mfp2$^{-/-}$ mice.* ***A)*** *Rhodopsin (red) staining did not reveal any differences between CT and Crx-Mfp2$^{-/-}$ mice for the rod photoreceptors.* ***B)*** *The cone photoreceptors were also unaltered. N=4/group. RPE: retinal pigment epithelium, POS: photoreceptor outer segment, PIS: photoreceptor inner segment, PR: photoreceptor, ONL: outer nuclear layer, OPL: outer plexiform layer, INL: inner nuclear layer, IPL: inner plexiform layer, GC: ganglion cell layer, RHO: rhodopsin, PNA: peanut agglutinin.*

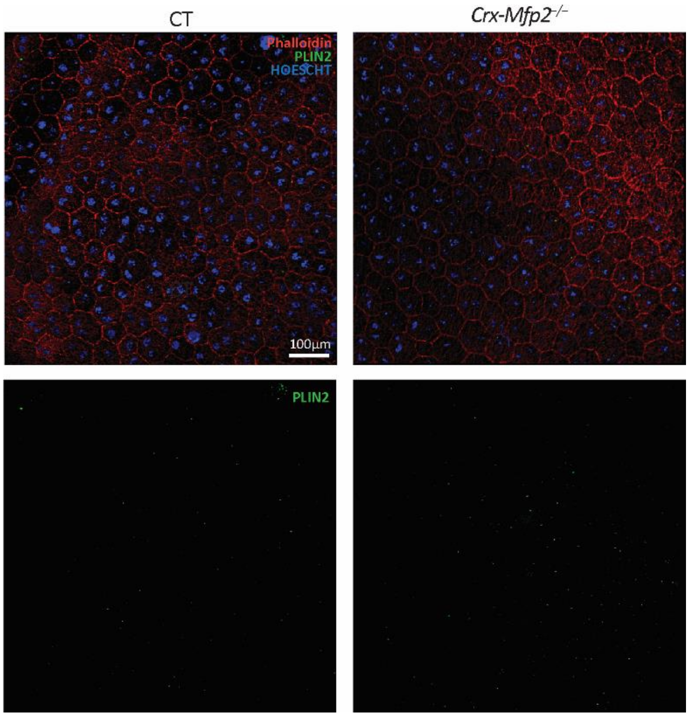

Figure S.4. *RPE health is not affected in 6-month-old Crx-Mfp2$^{-/-}$ mice. Double staining for phalloidin (F-actin cytoskeleton marker) (red) and PLIN2 (green) on RPE flatmounts revealed that the RPE maintained its hexagonal shape and there was no increase of lipid droplets. N=4/group. PLIN2: perilipin 2.*

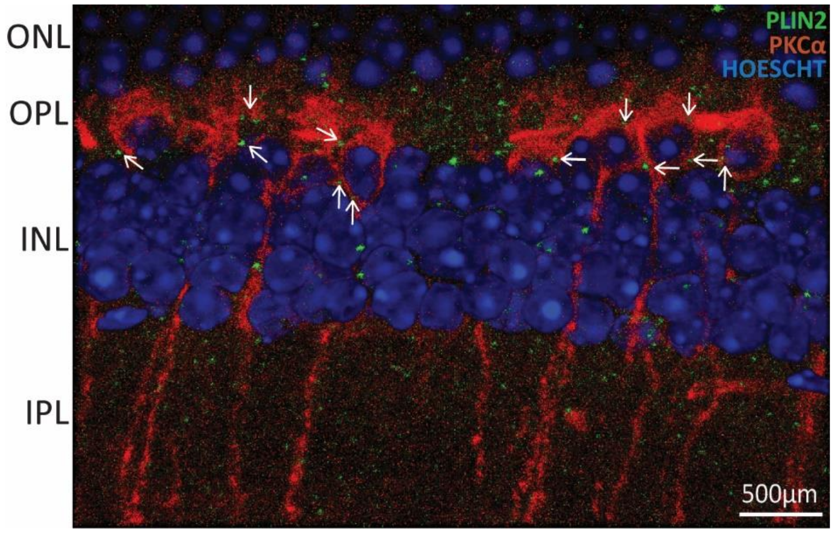

Figure S.5. *Lipid droplets accumulated in the rod bipolar cells of Crx-Mfp2$^{-/-}$ mice. Double staining of PLIN2 (green) with PKCα (red) revealed that lipid droplets accumulated in the rod bipolar cells of Crx-Mfp2$^{-/-}$ mice. N=4/group. ONL: outer nuclear layer, OPL: outer plexiform layer, INL: inner nuclear layer, IPL: inner plexiform layer, PKCα: protein kinase Cα, PLIN2: perilipin 2.*

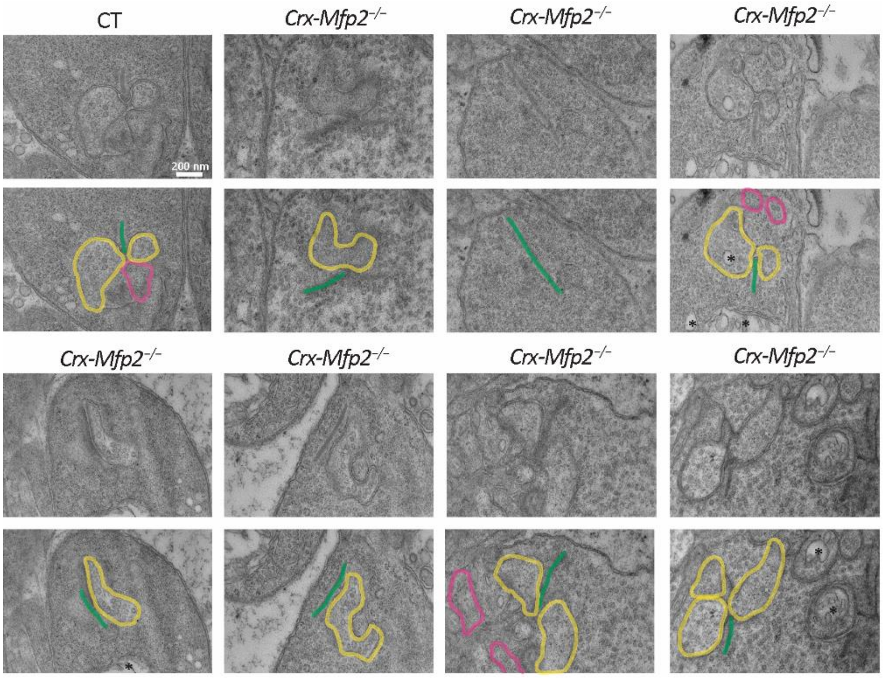

Figure S.6. *Additional TEM images of the photoreceptor ribbon synapse of 6-month-old Crx-Mfp2$^{-/-}$ mice. Ribbon (green), horizontal cell (yellow), bipolar cell (pink) and abnormal structures (*). Top and bottom panel are the same images. Bottom panels include interpretation. N=4/group.*

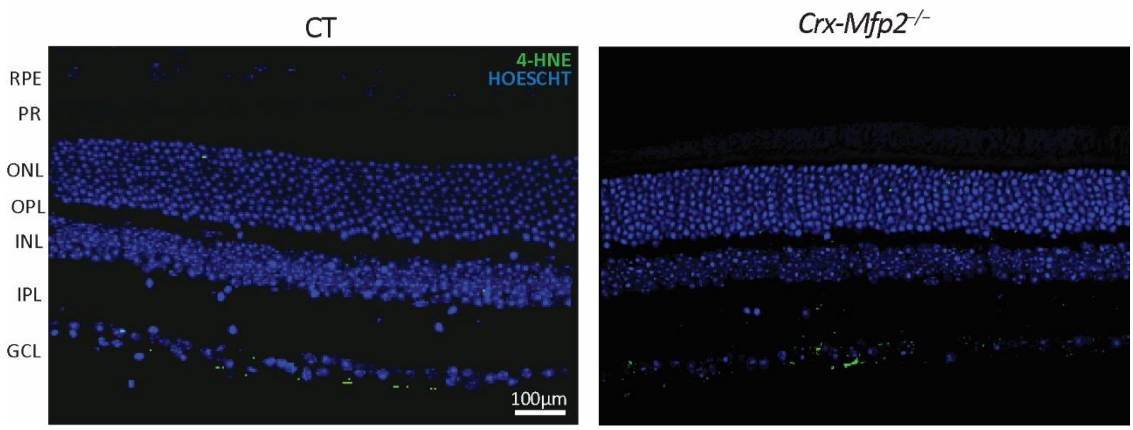

Figure S.7. *No increase of oxidative stress in the neural retina of Crx-Mfp2$^{-/-}$ mice. 4-HNE staining on the retina of 1-year-old Crx-Mfp2$^{-/-}$ mice did not reveal a significant change between CT and Crx-Mfp2$^{-/-}$ mice for 4-HNE expression. N=4/group. RPE: retinal pigment epithelium, PR: photoreceptor, ONL: outer nuclear layer, OPL: outer plexiform layer, INL: inner nuclear layer, IPL: inner plexiform layer, GC: ganglion cell layer, 4-HNE: 4-hydroxy-2-nonenal.*

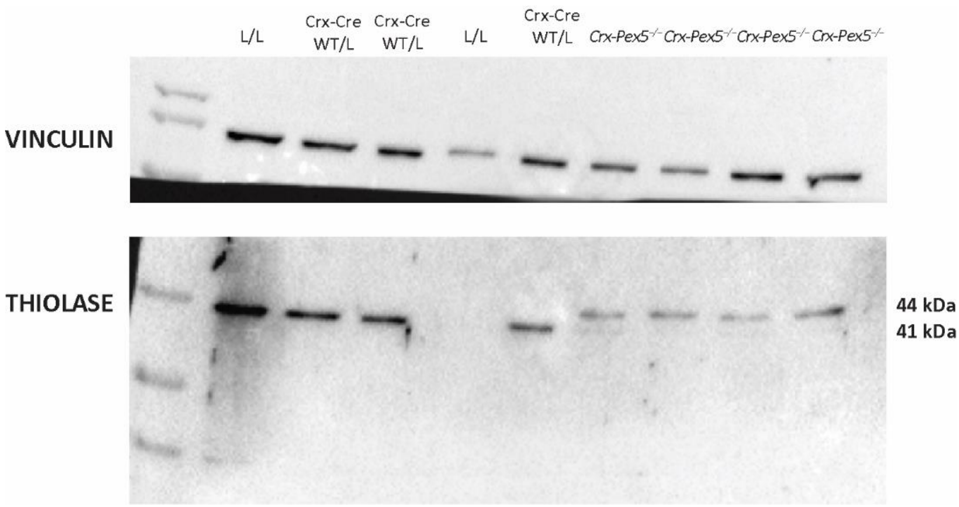

Figure S.8. *Full blot of the confirmation of Crx-Pex5 KO. Confirmation of Pex5 inactivation was evaluated via western blotting for thiolase. Normally, thiolase (44 kDa) is imported into peroxisomes via PEX5, where it is cleaved to 41 kDa. While the control mice show a band at 41 kDa, the Crx-Pex5$^{-/-}$ mice show a strong band at 44 kDa, suggesting impaired peroxisomal import. However, due to contamination with other interneuron cell types not under influence of the Crx promotor, a small band at 41 kDa appears in the Crx-Pex5$^{-/-}$ as well. Lane 4 was wrongly loaded, therefore not showing a band for thiolase and a very faint band for vinculin. Vinculin was used as loading control.*

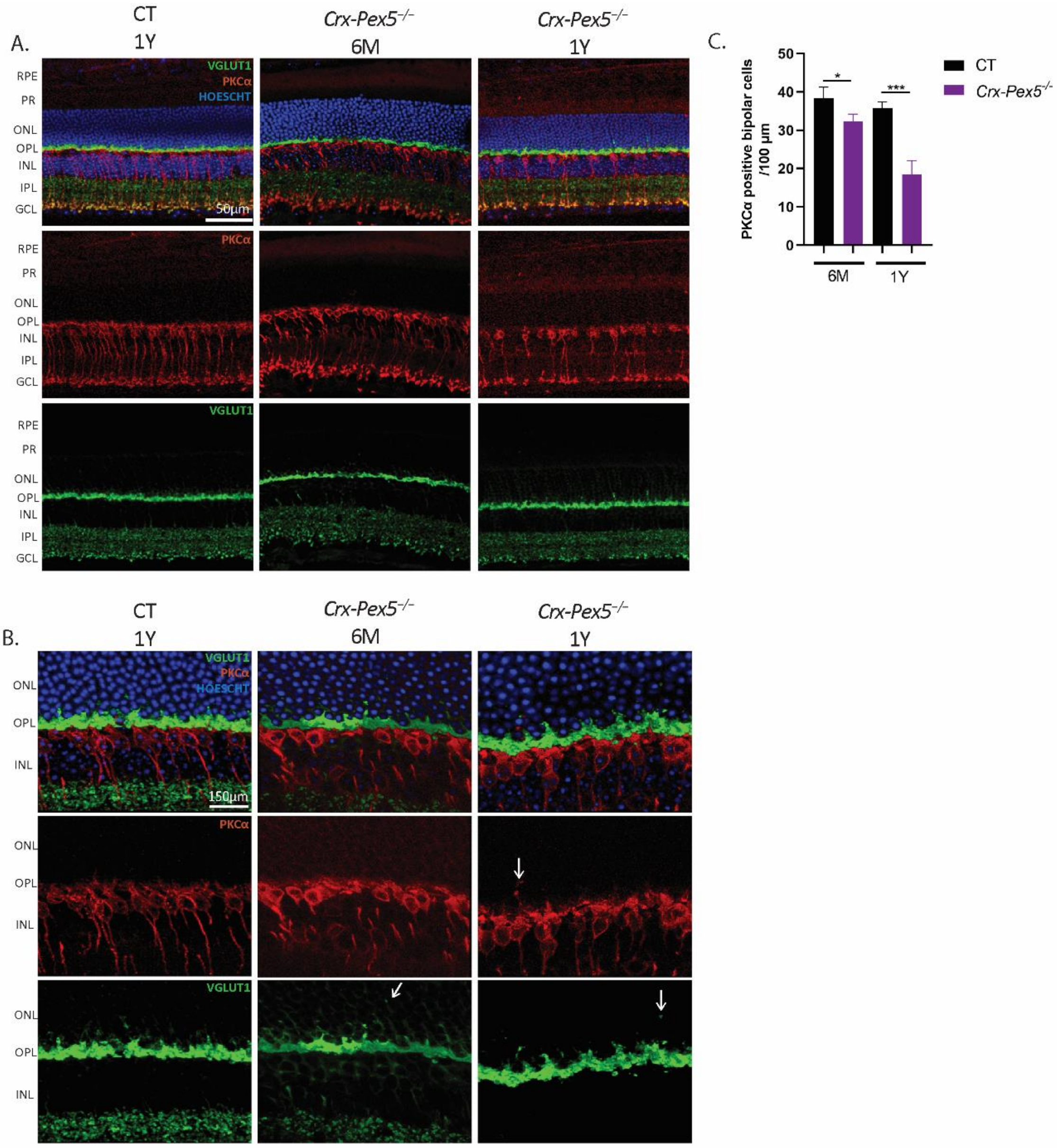

Figure S.9. *PKCα and VGLUT1 double staining in Crx-Pex5*$^{-/-}$ *mice reveals loss of bipolar cells and ectopic sprouting into the ONL.* ***A)*** *PKCα staining showed clear loss of bipolar cells in 1Y old Crx-Pex5*$^{-/-}$ *mice.* ***B)*** *Higher magnification images revealed sprouting of bipolar cells and VGLUT1 mislocalization into the ONL.* ***C)*** *Quantification of PKCα positive bipolar cells per 100 µm revealed a significant loss in both 6-month-old and 1-year-old Crx-Pex5*$^{-/-}$ *mice. N=4/group. Statistical difference based on unpaired t-test:* $*p < 0.05$, $***p < 0.001$. *Error bars indicate SD. RPE: retinal pigment epithelium, PR: photoreceptor, ONL: outer nuclear layer, OPL: outer plexiform layer, INL: inner nuclear layer, IPL: inner plexiform layer, GC: ganglion cell layer.*
